# Obituary

**Published:** 1893-04

**Authors:** 


### Obituary.

DR. W. W. ALLPORT—Died at his home, 69 Maple street, Chicago, of erysipelas, March 21, at 1:30 A. M. Aged sixty-eight years and ten months.

His sickness was of several weeks' duration, and very severe, he being for the most part during the last two weeks unconscious.

THE REGISTER will present a biographical sketch of Dr. Allport in the near future.

